# “We are responsible for the violence, and prevention is up to us”: a qualitative study of perceived risk factors for gender-based violence among Ethiopian university students

**DOI:** 10.1186/s12905-019-0824-0

**Published:** 2019-11-06

**Authors:** Michelle R. Kaufman, Ashlie M. Williams, Graziele Grilo, Christina X. Marea, Fasil Walelign Fentaye, Lakew Abebe Gebretsadik, Shifera Asfaw Yedenekal

**Affiliations:** 1 Department of Health, Behavior & Society Johns Hopkins Bloomberg School of Public Health, 624 N. Broadway, Baltimore, MD 21205 USA; 2 Johns Hopkins School of Nursing, 525 N. Wolfe Street, Baltimore, MD 21205 USA; 3 Department of Public Health, Wollo University, P.O. Box 1145, Dessie, Ethiopia; 4 Department of Health, Behavior and Society, Faculty of Public Health, Institute of Heath, Jimma University, Jimma, Ethiopia

**Keywords:** Adolescents, Ethiopia, Interpersonal violence, Intimate partner violence, Gender-based violence, Physical abuse, Sexual violence, Youth, University

## Abstract

**Background:**

There is a high prevalence of gender-based violence (GBV) victimization among young Ethiopian women, including in universities, where female enrollment is low but growing. Understanding factors contributing to GBV in this context and students’ perspectives on gender, relationships, and interpersonal violence is essential to creating effective interventions to prevent GBV and support female students’ rights and wellbeing.

**Methods:**

In-depth interviews (IDIs) and focus group discussions (FGDs) were held with male and female students (male IDI *n* = 36, female IDI *n* = 34, male FGD *n* = 18, female FGD *n* = 19) and faculty and staff (FGD *n* = 19) at two Ethiopian universities. Audio recordings were transcribed and translated into English. Transcripts were coded thematically to identify key factors contributing to GBV and provide narratives of students’ experiences.

**Results:**

GBV against female students was a salient issue, including narrative accounts of harassment, intimidation, and physical and sexual violence on the university campuses and the towns in which they are located. Reported risks for GBV included receiving academic support from male peers, exercising agency in relationship decision-making, having a negative self-concept, belief in stereotypical gender expectations, and engaging in transactional sex and/or substance use. While students recognized these risk factors, they also suggested GBV may be the result of females’ “improper” behavior, attire, use of males for personal gain, or personal failure to prevent violence.

**Conclusions:**

GBV is a serious issue in these two Ethiopian universities, creating a tenuous learning environment for female students. Programs are needed to address areas of vulnerability and negative attitudes toward female students in order to decrease female victimization.

## Background

Gender-based violence (GBV) in Ethiopia is a serious public health concern. The 2005 World Health Organization Multi-Country Study on Women’s Health and Domestic Violence against Women [1] found that 71% of ever partnered women in Ethiopia had experienced physical or sexual violence by an intimate partner. Intimate partner violence (IPV) and other forms of GBV in Ethiopia have adverse health impacts. Ethiopian women who experience IPV are almost twice as likely to have HIV [2]. Female undergraduate students who experienced IPV or other forms of GBV in a study in Awassa, Ethiopia were up to four times more likely to suffer depressive symptoms compared to those who had not [3], and a community-based study in Agaro Town found that suicidal ideation was three times more likely among reproductive age women who had experienced sexual abuse compared to those who had not [4].

While women’s educational status may be protective against GBV [5], university-affiliated women in Ethiopia face persistent risks for GBV victimization and experience it at high rates. Despite Ministry of Education affirmative action policies, the proportion of female university students, staff, and women in leadership positions is low [6]. Female students represented only 32% of all undergraduates in 2016 [6], and female instructors comprised 11% of university teaching staff in 2014 [7]. Quantitative studies among undergraduates in Awassa found that 40% of females reported having experienced GBV [8], and 24% and 17% of males reported having perpetrated physical or sexual violence, respectively, in the current academic year [9].

Given high rates of GBV in Ethiopia generally and among university-aged women in particular [1], as well as the increasing numbers of women attending universities in Ethiopia [6, 7], prevention efforts are necessary to address this public health concern. While a small body of quantitative research has reported on prevalence of and risk factors for GBV among university students in Ethiopia [8–10], no known study has qualitatively explored this population’s perceptions of drivers of GBV or their experiences of this type of violence. Through qualitative research, the goal of this study was to identify factors students believe contribute to GBV risk on their campuses. In doing so, we offer researchers and practitioners the opportunity to ensure that future interventions are tailored to students’ needs and grounded in students’ voices.

## Methods

This study explored narrative accounts of Ethiopian university students’ perceptions and experiences of romantic relationships, sexual behavior, gender norms, and GBV. By analyzing student beliefs of the causes of GBV, the study sought to complement existing quantitative data on prevalence of [8–10] and attitudes toward [11, 12] GBV and to inform future prevention efforts.

The Jimma University, Wollo University College of Medicine and Health Sciences, and the Johns Hopkins Bloomberg School of Public Health Institutional Review Boards granted ethical approval.

### Setting

Qualitative data were collected at two Ethiopian universities in 2016.

#### University A

University A is located southwest of the capital of Ethiopia, Addis Ababa. It was established in 1999 by amalgamating several other colleges, the oldest founded in 1952. As of 2017, the university had over 16,000 undergraduate and 2300 graduate students, primarily of rural origin, across four campuses. Females comprise approximately 35% of undergraduate students.

#### University B

The Government of Ethiopia established University B in 2007 in the north-central part of the country on two campuses close to a city center. In 2017, there were nearly 26,000 undergraduate and over 1700 graduate students, mostly from rural areas. Females comprised 29% of undergraduate students.

### Participants

Student recruitment for interview participation was through convenience sampling via announcements and flyers. Male and female undergraduate students ages 18 years and older were eligible to participate in in-depth interviews (IDIs; *n* = 34 for females, 36 for males) and focus group discussions (FGDs; 2 student FGDs per university, total *n* = 19 for females, 18 for males). At each university, one FGD was also held with university faculty and staff (total *n* = 19), who were recruited through targeted sampling of participants who could offer insight into GBV issues on campus. See Table 1 for participant details.

**Table 1:** Participant Demographics

	IDIs (*n* = 70)		FGDs Students (*n* = 37)		FGDs Faculty (*n* = 19)	
	Women	Men	Women	Men	Women	Men
Total Participants	34 (49%)	36 (51%)	19 (51%)	18 (49%)	8 (42%)	11 (48%)
Mean Age (SD)	21.5 (1.8)^a^	21.6 (2.2)^b^	22.5 (1.5)	21.8 (1.9)	26.6 (3.9)	31.2 (5.4)
Mean Years (SD) of University Education (Students)	2.5 (1.1)^c^	2.5 (1.2)^a^	3.3 (1.3)	2.8 (1.3)	–	–

^a^ 3 missing values

^b^ 2 missing values

^c^ 4 missing values

### Data collection procedures

Local public health professionals not affiliated with the universities under study facilitated all data collection in April 2016. The facilitators received training on qualitative data collection procedures, the study purpose, and ethical treatment of participants with an emphasis on the sensitive nature of GBV research. In recruitment material and during the consent process, the purpose of the study was described to participants as “to learn more about what romantic and sexual relationships are like for students at this university.” All study materials indicated that participants would be asked questions related to romantic relationships, sexuality, HIV, or relationship violence (we did not specify violence against women only).

Interviews were conducted with a field team member of the same gender unless the participant indicated he/she would prefer to interview with someone of another gender (which occurred in six female interviews). To start, the facilitator explained the study purpose and procedures to prospective participants and obtained signed informed consent. All interviews were conducted in the participant’s preferred language (Amharic, Afan Oromo, or English) and audio recorded with permission of the participant. IDIs took 45 to 90 min each, and FGDs took 90 to 120 min each. All discussions were held in private locations on each respective campus. Audio recordings were transcribed verbatim and translated into English by local native speakers.

Facilitators used semi-structured interview guides for all IDIs and FGDs developed for this study by the Ethiopian research team and the lead authors (see Additional files 1, 2, 3, 4 and 5). They asked participants open-ended questions about the nature of romantic and sexual relationships among students, including student relationships with teachers and individuals not affiliated with the university. Participants were asked to give their own definitions of GBV and IPV and their understanding of the causes of these types of violence; facilitators did not offer a definition of these terms at any time. Subsequent interview questions relating to GBV and IPV focused on female students’ experiences. Participants were invited to describe whether female students at that university experience gender-based violence on or near campus (including personal experiences), ways in which violence affects female students’ health, and what on-campus resources are available to female students who have been affected by GBV.

### Data analysis

Following translation of the transcripts into English, thematic coding was conducted using Atlas.ti version 8. A member of the research team developed a preliminary codebook after reading the transcripts. Two team members then each coded two transcripts using the preliminary codebook. After discussing the coding of these transcripts and differences in interpretation of the code definitions, the codebook was refined. Three female members of the research team, all graduate students, double-coded all remaining transcripts. They again compared codes and discussed discrepancies in order to reach at least 85% agreement. Local investigators in Ethiopia were consulted for clarification on themes arising from the data, but they did not make any requests to remove or modify any of the identified themes.

Ultimately, the analysis identified thirteen themes relating to GBV, sexual risk, and student experiences. This paper focuses on four of these themes. While the results presented below are primarily drawn from participants’ responses to interview questions dealing with GBV, the full content of the interviews informed the analysis. For example, the data on the nature of romantic relationships on campus provided context for students’ discussion of GBV. Subsequent papers explore other themes in greater depth [13] (for an analysis of those relating to the intersection of sexual risk behavior and GBV). The research team also noted points of agreement or disagreement between male and female students; between students, faculty, and staff; and between in-depth interview and focus group participants. This served to develop a fuller understanding of campus community members’ attitudes and beliefs relating to gender and GBV.

## Results

The four themes reported on here include: (a) experiences with GBV, (b) perceived risk factors for victimization, (c) assigning blame, and (d) supports for victims. See Table 2 for a summary of themes and key findings.

**Table 2:** Key Findings by Theme

Themes	Key Findings
Experiences with GBV	Harassment, threats, and intimidation reportedly occur frequently on both campuses, especially in common places such as the library.
	Female students from both universities reported that IPV and coerced sex are common in students’ relationships.
	Cases of male teachers manipulating female students’ grades in exchange for sex were reported at both universities.
Risk factors for victimization	The low social status of female students and their perceived academic inferiority contribute to their risk of victimization by male peers and faculty.
	Traditional relationship dynamics, such as the obligation for females to have sex, may result in GBV if a female student refuses to have sex or enter a relationship with a male.
	Female students’ engagement in relationships with males in order to obtain financial support may result in sexual coercion, particularly in combination with substance use.
Assigning Blame for Perpetration	Male participants indicated that GBV occurred because female students use male students for personal gain.
	Both male and female participants saw women wearing certain clothing as contributing to GBV and expressed the belief that some men are unable to control their sexual impulses.
	Both male and female participants accused female students of putting themselves in harm’s way.
Victim Support	Peers were identified as a main source of support among victims of GBV.
	Campus resources, including a Gender Office, campus police, and mental health services were often viewed as ineffective.
	Male students expressed resentment for affirmative action policies and other supports for female students.

### Experiences with GBV

Few participants shared firsthand accounts of GBV, but many told of female peers who had experienced GBV in various forms, including harassment and intimidation, IPV, and sexual coercion by faculty in exchange for grades.

#### Harassment and intimidation

Participants reported that female students are harassed frequently on campus and feel uncomfortable in common spaces such as the library as a result. Participants also reported that female students experience threats and intimidation by males, including threats of physical violence. Their female peers were reported to have entered relationships out of fear of violent retaliation if they refused male advances:

“One of the males outside of campus asked her for a relationship. The man was aggressive, and fearing him she started a relationship with him. He is muscular and seemed like a boxer, and he even beat her.” (female student IDI, year 2, University A)

“There was a female student, and a guy from outside campus was asking her for love [ … ]. He started to meet her by force, telling her that he knew teachers, and if she refused him he would harm her.” (male faculty/staff FGD, University A)

#### IPV

IPV was prominent in participants’ discussion of GBV on campus. Accounts of IPV described overt physical abuse, coerced sex, and controlling behaviors such as restricting a female partner’s interactions with other males or her cell phone use:

“When I was a student, I knew a guy who had crossed into the girls’ dorm and beat his lover and was suspended for two years. I know about four girls who were victims of beating by their love partners.” (female faculty/staff FGD, University B)

#### Sex exchanged for grades

Participants described cases in which faculty manipulated female students’ grades in exchange for sex:

“Teachers give you bad grades and want you to come negotiate [ … ] I know a girl who was called to the teacher’s office who showed her what she scored, which was good, but she refused to fulfill his [sexual] demands. Then he posted ‘Incomplete’ when the grades were announced.” (female student IDI, year 4, University B)

Only female students and one female faculty member discussed sexual coercion by faculty at University B. At University A, male students and one male faculty member agreed with female participants that female students indeed face grade manipulation in exchange for sex.

### Perceived risk factors for victimization

Students and faculty noted several factors that place female university students at risk for GBV, including the low social status of females on campus and in Ethiopian society, endorsing “traditional” relationship dynamics, and transactional sex—particularly in combination with substance use.

#### Low social status of female students

Students reported both male and female students intentionally seek relationships predicated upon the male providing academic assistance to the female, as female students are viewed as requiring more academic support than male students. Some males, they said, expect sex in return for the assistance. Students believed that this perceived obligation to have sex after receiving academic help leads to GBV:

“Female students consider themselves poor academic performers; even the male students perceive them like that. For this reason they start a relationship. When female students refuse sex, the violence happens.” (female student IDI, year 3, University A)

Participants believed female first-year students, students from rural areas, and students with low self-confidence are most vulnerable to this dynamic.

Gender norms and notions of female inferiority contribute to other forms of GBV on campus. Both male and female participants reported that Ethiopian culture supports widespread beliefs about male superiority:

“It could be the culture and the way we are brought up, but sometimes it could also be the perception of women themselves because even when their rights are respected, they feel inferior or they don’t have self-esteem. Men are seen as confident in the culture, which makes them feel extreme self-esteem, which even encourages them to see women as inferior.” (male student IDI, year 2, University B)

Students reported females generally have low social value in Ethiopian society. This devaluation, reinforced by female students’ minority status on campus, was reported to be a cause of GBV in the university context:

“The cause [of GBV] starts from home because boys don’t [do household] work if there is a girl in the house. There are more boy students than girls. So the boys feel superior mentally. Even when they talk, if a girl gives responses against a boy’s wish, the boy may use [physical] force on her.” (female student IDI, year 3, University A)

#### Traditional relationship dynamics

Students reported that traditional relationship dynamics place female students at risk for violence. Several female participants described having sex with male partners as “mandatory” because of these traditional schemas:

“[Male students] think having sex is mandatory in a relationship. If she says no to his request, he will commit violence.” (female student IDI, year 3, University A)

These traditional dynamics also lead some students to not classify IPV as such:

“I believe that, though I have the right to refuse his request, because I am female I want to obey his interest. Therefore, thinking this I do not take it as violence. This is a normal condition, as it comes from our grandparents and families.” (female student IDI, year 2, University A)

Conversely, females seem to face increased risk of GBV when they attempt to exercise agency in relationship decision-making, violating traditional gender roles:

“Since [males] know that some girls are easygoing, when you resist them, they [males] say, ‘Who does she think she is? Isn’t she a woman?’ and they start to attack you.” (female student IDI, year 2, University B)

GBV was reported to occur when females turn down males’ invitations to begin romantic relationships. One told of her personal experience repeatedly refusing a male peer’s requests:

“One day, I had an exam, and he was waiting for me along the street by the examination room. And he tried to hold my hand as usual and talk to me, and I told him that I didn’t have time to talk with him as I was going to take an exam. But, he was not accepting what I told him. He even slapped me, and [ … ] I faced nasal bleeding.” (female student IDI, undisclosed year, University A)

Risks of violence occur at multiple stages in a relationship. Many participants reported that males sometimes become violent when female students attempt to leave their relationships.

#### Transactional sex

Certain behaviors of female students were also viewed as increasing their risk for GBV, including transactional sex for financial support. Male and female participants reported that female students sometimes engage in relationships with men outside of campus in order to support themselves financially during their studies. These “sugar daddies” may become violent or sexually coercive.

“There are female students who want to get money from other people through sexual activity. If female students receive incentives for sexual activity, it becomes difficult to terminate the relationship. Most of the time graduating class female students become sexual partners with individuals who are financially capacitated. There are brokers that link those female students with businessmen.” (male faculty/staff FGD, University A)

#### Substance use

Participants reported that alcohol-related GBV is common in relation to transactional sex and when students go into town for fun. Students who visit nightclubs and stay in hotels after becoming intoxicated were seen as particularly vulnerable to GBV. Some told stories of females who went out on the town and were subsequently raped:

“[In a nightclub, a male student and a female student were] taking beer little by little and [...] he got drunk. Finally, the time was over; they realized it was impossible to enter the university at that time. They agreed to rest in a hotel separately [in separate beds]. However, there was only one bed left [...]. They agreed to sleep in the bed together, but he ultimately forced her and took her virginity.” (female student IDI, year 3, University A)

“Once I came across a girl student in a taxi who had been made drunk and raped; they just put her in a taxi and left. She was crying without conscious mind since she was drunk. [ … ] Since she failed to keep her balance in walking, I had to help her to the dorm. Then on the way she told me that she was a freshman, and some [male] students invited her to go out and relax. They got her drunk, then took her to a bedroom.” (male student IDI, year 5, University B)

### Who is to blame for GBV?

Participants tried to assign blame for GBV occurrences on university campuses, listing females’ behavior, dress, and lack of personal prevention measures as causes of violence.

#### Female behavior

Participants at University A felt that GBV was the result of females being involved with multiple males:

“If a female student has multiple sexual relationships with male students, she loses respect from others. They insult her for misbehaving and not acting as a well-mannered woman. [ … ] If she gives some unnecessary response, they spit in her face.” (female student IDI, year 3, University A)

Male students, also at University A, justified GBV by arguing that if a female student receives favors or support from a male student, he can expect sex with this female. For example, two recalled a male student who expected sex from a female peer whom he helped to cheat on exams. After the female refused to have sex with him, he beat her severely and was briefly sent to prison. One participant offered his opinion on this incident:

“Many male students were offended by the actions of female students, especially after we [learned] that a female student complicated the life of a genius student and let him be imprisoned. [ … ] If she told him from the beginning [that she was not interested in sex], he may not have gotten hurt.” (male student IDI, year 3, University A)

#### Female dress

Students and faculty also blamed GBV on females’ wardrobe; women who wear trousers or revealing clothing were seen as inviting violence. Male and female participants both reported a belief that some males have difficulty controlling sexual impulses when they see women wearing such clothing:

“Sometimes it is the women themselves who make you cause such abuse on them [ …] you know when the man is drunk and the girl’s dressing style is seducing, it may push him to rape her.” (male student IDI, year 2, University B).

#### Putting oneself in harm’s way

Participants argued that female students should do more to protect themselves from GBV, blaming the victim for her experience. One male faculty member clearly engaged in this victim blaming, although this attitude came up repeatedly:

“Last year a man invited a female student outside the campus at 10:00 at night and beat her, which affected her eyes seriously. But whose fault is this? She is the one who went out at night willingly and entered his house.” (male faculty/staff FGD, University B)

Female participants also emphasized females’ personal responsibility to avoid situations that put them at risk for GBV:

“The other reason [for GBV] is that females [go to] risky places such as outside the dorm and campus at risky times like overnight [ … ] [Gender] Equality is not about [going to] risky places [ … ], rather to be smart and think more wisely than males. Males [can go] every place they want, like dark time, etc., since they trust their forces, and we females should think before them and pass them wisely.” (female student FGD, year 2, University A).

“I believe that we are responsible for the violence and prevention is up to us. We have to improve our behavior and we have to inform responsible bodies when there is violence.” (female student IDI, year 3, University A)

Male and female students and faculty at both universities tended to talk in terms of a victim being at least partially responsible for a GBV experience.

### Victim support

Despite the reported widespread incidence of GBV on campus, students and faculty noted sources of support for victims, including peers and campus-based resources.

#### Peer support

Female students seemed to rely primarily on peers for support after experiencing GBV. They reported walking in groups to avoid GBV and shared examples of intervention by peer bystanders; however, peers do not always step in. A student described his experience witnessing the abuse of a female peer:

“We went to her and picked her up. She was bleeding from her nose, but no one tried to stop him, everybody was silent.” (male student IDI, year 3, University A)

While female students said victims might disclose GBV incidents to close friends, males said it is uncommon for them to discuss GBV prevention with their male peers.

#### Campus resources

Each university’s Gender Office and the campus police were discussed sources of prevention and recourse for GBV, though there were students who were unaware of the existence of a Gender Office on campus. Students were doubtful of these avenues of recourse, often deciding not to report incidents. Females feared gossip among peers or violent retaliation if they brought attention to the issue:

“They can also report to legal bodies – police, Gender Office and so on – but usually female students don’t do this because of fear of the consequence by other cliques, even when the perpetrator is not around or punished for a year or so.” (female student IDI, year 3, University A)

Students also did not believe anything would come of reporting, a theme linked to the belief that blame lies with the victim:

“First of all, no one reports the case, plus what evidence can I bring? Thirdly, even if I report it, the answer will be ‘Why did you go to the town at night?’ and consider it as you did it in your own interest.” (female student IDI, year 1, University B)

When victims do report incidences of GBV, students and faculty members claimed the university does not take substantive action, or the process takes too long. They felt this may increase students’ vulnerability to manipulation by teachers:

“New students to the university come with a mind that if any teacher forces them to have sex, there is no way to say no.” (male student IDI, year 5, University B)

Students also alleged that some campus police turn a blind eye to certain male students and claimed that some police, too, have perpetrated violence against female students. Faculty FGD participants did describe cases in which they intervened upon students’ reports of GBV, however.

Beyond these bodies, students described a need for further campus resources. Participants believed psychological services were lacking for students who had experienced GBV. They also said that additional financial support for female students could prevent peers from engaging in transactional sex. They indicated a need for on-campus recreation facilities to help students avoid placing themselves at risk; off-campus activities were perceived as more risky because of the prevalence of substance use and the presence of “sugar daddies”. Male students, however, expressed resentment of the support services and affirmative action policies benefitting females already in place at their universities:

“I don’t have words, girls are respected because of affirmative action. I mean if we do equal in any assignment, girls at least will earn 10 more points. What they have to do is participate in class, laugh by sitting in front towards the teacher. The so-called ‘affirmative action’ is rather making them lag behind.” (male student FGD, year 3, University B)

The question of additional support services for female students seemed to trigger annoyance among some males who expressed the belief that females are already receiving more support than is fair compared to male students.

## Discussion

This study suggests that GBV is common in the lives of undergraduate students at two Ethiopian universities and identifies perceived factors associated with victimization. Students and faculty agreed that female students face a variety of forms of GBV, including harassment, intimidation, physical violence, and sexual violence by male partners, peers, faculty, and community members. This is the first known study in Ethiopian universities to qualitatively explore perceived drivers of GBV.

The findings of the current study support those of quantitative studies on the prevalence of GBV and IPV in Ethiopia [1, 3, 5, 8–10, 12, 14–17]. Some female students in the current study entered relationships with male peers as a result of—or in order to avoid—harassment, threats of violence, and intimidation. Those who rejected male requests for romantic relationships were sometimes physically assaulted, while others experienced violence when ending a relationship. These reports highlight two sides of this complex issue. First, fear of violence contributes to unequal relationships in which females have reduced decision-making power about sex and whether to leave the relationship, which is exacerbated by financial insecurity, as reports of “sugar daddy” relationships highlight. This renders female students more susceptible to sexual coercion and violence. Quantitative studies [1, 3, 5, 12, 14–17] in Ethiopia have found women’s reduced relationship decision-making power to be significantly associated with experiences of IPV, as have studies conducted in other contexts [5, 17]. Second, females who exercise agency or demonstrate confidence and assertiveness may face violent retaliation, both within and independent of a romantic relationship, for their violation of traditional gender norms.

Potential interventions to address unequal relationships and backlash against female agency would need to go beyond typical behavioral interventions that promote healthy relationships to address the wider context of misogyny seen on these university campuses. Gender-transformative interventions, which promote gender-equitable relationships by shifting gender norms, have successfully reduced GBV in other contexts [18, 19]. A 2007 WHO-supported review found that gender-transformative interventions with males are more effective in reducing violence than interventions that accommodate or ignore gender norms [20], though there is a need to test such interventions in the Ethiopian context. The extent to which cultural beliefs appear to normalize and reinforce GBV in this study highlights the importance of clearly defining GBV in these interventions.

Beyond unequal relationship dynamics and backlash against female agency, narrative reports of GBV further illustrate how the perceived academic inferiority of female students increases risk of GBV, especially given their low numbers on campus. In our findings, this is seen when male faculty manipulate grades as well as when female students seek or accept academic help from males, who may view female students as weaker academically and resent affirmative action policies. These views echo findings from qualitative research in Tanzania, which found that students believed that the existence of affirmative action policies for female students proved females were under-qualified to attend university [21]. In the current study, attitudes of female inferiority and concomitant resentment create a climate in which some male students and faculty feel justified in expecting, pressuring, or forcing female students to have sex with them in exchange for academic support. Female students have limited avenues for avoiding such situations because males comprise a majority of the student body and faculty. Practical responses to this issue could include expanding female students’ options for whom to ask for academic support, such as by creating formal tutoring systems or study groups, as well as greater accountability for perpetrators. Eliminating issues related to beliefs of female inferiority will require broader promotion of gender-equitable attitudes to increase males’ support for programs that promote female students’ education.

This study suggests possible ways to target future GBV prevention interventions in the Ethiopian university setting. Students perceived unequal relationship dynamics to be especially common among first-year females, who may have low confidence navigating the university environment, and gave narrative reports of GBV perpetrated against first-year students by upper class students. Quantitative research at Wolaita Sodo University in Southern Ethiopia similarly found that experiences of sexual victimization and harassment were most prevalent among first-year females [22]. This indicates the need for interventions at the time of matriculation. Among first-year females, promoting a stronger self-concept through same-gender role models and mentors could help to increase their confidence in navigating their urban university and resisting sexual coercion [10]. Equipping first-year females with the ability to recognize and respond to risk for violence could also reduce GBV, and interventions could be adapted from other contexts [23]. As the current study found that greater confidence and agency could also increase females’ vulnerability to violence – a backlash effect – it is vital for interventions to concurrently target males. Interventions aimed at male students must address beliefs about obligations to have sex and harmful gender schemas. While upper class students were reportedly more likely to commit violence in this study, it may be more feasible to engage first-year males, with mandatory trainings included as part of the university orientation and mentorship programs with older classmates or faculty who model gender-equitable behaviors and positive masculinity. Despite the lack of examples of the use of such interventions in the university context, interventions adapted from other school-based efforts to engage males in GBV prevention [24, 25] could be developed and tested in these universities.

Despite their recognition of the gender norms and structural factors that contribute to GBV, students in the current study blamed female victims. Students also believed that peers, university faculty, and staff would blame the female victim for any GBV she experiences. This is consistent with findings that many university-aged men and women believe wife beating is justified as a response to or punishment for female behaviors [26, 27]. Victim blaming indicates that gender stereotypes and double standards persist, contributing to a climate in which many students are not surprised by occurrences of GBV. Preventing GBV thus requires not only increased support and security for female students, but also shifts in attitudes and beliefs about gender and human rights among male and female students, faculty, and staff.

Previous quantitative research on risk factors for GBV victimization among Ethiopian university students identified substance use as a key predictor [12, 28]. The qualitative findings presented here support the finding that substance use is an important risk for GBV, but that this often occurs off campus. On-campus measures such as penalizing alcohol use may thus have unintended effects. As student narratives suggest, students may respond to such policies by staying in off-campus accommodations, where sexual violence more often occurs. The assignment of blame to the victim that occurs when she has engaged in substance use may also limit victims’ belief in their ability to hold perpetrators accountable, as students reported. Trainings and policies intended to prevent substance use and GBV must take these complexities into consideration.

In light of reports of GBV, the university can take immediate steps to improve student safety. Bystander intervention trainings could build upon victims’ reported reliance on peers for support. There is evidence that these programs have worked in the North American university context [8, 9], but they have yet to be tested in or adapted to the Ethiopian context. A major hindrance to their effectiveness in Ethiopian universities is weak enforcement or lack of policies such as terminating professors who have sex with students. Indeed, this study found that the perpetration of GBV by male students and faculty is not a hidden issue on these campuses, yet it may go unpunished. Strengthening support and accountability structures, particularly by addressing student concerns about privacy and accountability in reporting incidents of GBV, must therefore occur simultaneously with violence prevention. Safety promotion could also include a more effective security presence on campus – which was identified by student participants as lacking.

This study has limitations. There is a potential for bias in the translation of the interview transcripts, as university faculty conducted some translations (others were conducted by a hired translator not connected with the study) and may have had an unconscious bias. The interviews were also translated into English without subsequent back-translation to the original language to verify meaning. Caution should also be used in drawing conclusions about one university compared to the other, or one gender of participants versus the other, given the convenience sampling frame. The data presented here represent the views of individuals at two Ethiopian universities and may not be representative of other universities, particularly those serving different regions or demographics. Given the qualitative methodology used in this study, the experiences that research participants reported have not been validated. The insights offered by accounts of GBV in this study, however, underscore the importance of qualitative research in understanding the causes of GBV and opportunities for prevention, and the need for additional studies among university populations in Ethiopia.

## Conclusions

This study adds essential insight to the existing body of research on GBV in Ethiopia. Beyond prevalence estimates and analyses of demographic characteristics associated with risk, the themes identified here represent current social dynamics operating within the context of the university and student relationships. The behaviors and attitudes discussed suggest actionable areas for improvement in universities’ efforts to support female students and prevent GBV through interventions. By providing female students with effective support and security and promoting gender equitable attitudes among students, faculty, and staff, the university can be a stepping-stone—rather than a hurdle—toward females’ realization of their full potential.

## Supplementary information

**Additional file 1.** in-depth interview guide for female students.

**Additional file 2.** in-depth interview guide for male students.

**Additional file 3.** focus group guide for female students.

**Additional file 4.** focus group guide for male students.

**Additional file 5.** focus group guide for faculty/staff.

## Data Availability

The data analyzed in the current study are not publicly available to protect the privacy and anonymity of the participants.

## Abbreviations

FGD: Focus group discussion
GBV: Gender-based violence
IDI: In-depth interview
IPV: Intimate partner violence
