# Supplementary material for: “We are responsible for the violence, and prevention is up to us”: a qualitative study of perceived risk factors for gender-based violence among Ethiopian university students
Source: BMC Womens Health. 2019 Nov 6;19:131. doi: 10.1186/s12905-019-0824-0 (PMC6836646; doi:10.1186/s12905-019-0824-0)
Supplement: Supplementary file 2 — Additional file 2. in-depth interview guide for male students. [file 12905_2019_824_MOESM2_ESM.pdf]

In-Depth Interview with MALE Students

Thank you for agreeing to speak with me. As I said during the consent process, I am going to ask you several questions, some of them very personal and sensitive in nature. I ask that you please be honest in your answers. If you feel uncomfortable at any time, you can decide not to answer a particular question, or we can stop all together. Are you ready to begin?

1. Let's start by talking about romantic relationships among university students. Tell me about relationships between men and women.
2. Tell me about the sexual behaviors of students.
3. Now let's talk about how female students are treated on campus.
4. How do you define gender-based violence (GBV)?
5. How do you define intimate partner violence?
6. Do these types of violence happen to female students at this university? Why?
7. What resources are available to female students on campus if they experience violence?
8. What happens to men at this university if they commit violence against women?
9. Does violence or the threat of violence affect female students' sexual health? How?
10. Do you know anyone who experienced or committed this type of violence? Without using any names, tell me what happened to them.
11. Have you ever discussed preventing violence against women with other male students? What did you talk about?
12. Have you ever experienced this type of violence since you've been at the university? Please tell me what happened.
13. If incoming freshmen students were to receive training on sex and HIV, what would it look like?
14. What if incoming freshmen students were to receive training on gender-based violence, what would it look like?
15. Is there anything you wish the university would do differently for students regarding STIs/HIV? For male students in particular?
16. Is there anything you wish the university would do differently for students regarding GBV?
17. Is there anything else you would like to tell me about what we discussed today, or anything else you think I should know?
18. Do you have any questions for me?

Thank the respondent for his time, provide the resource guide, and deliver payment.
