# Supplementary material for: “We are responsible for the violence, and prevention is up to us”: a qualitative study of perceived risk factors for gender-based violence among Ethiopian university students
Source: BMC Womens Health. 2019 Nov 6;19:131. doi: 10.1186/s12905-019-0824-0 (PMC6836646; doi:10.1186/s12905-019-0824-0)
Supplement: Supplementary file 4 — Additional file 4. focus group guide for male students. [file 12905_2019_824_MOESM4_ESM.pdf]

Focus Group Discussion with MALE Students

Thank you for agreeing to participate in the discussion. As I said during the consent process, I am going to ask you several questions about sex and violence on campus, but I will not ask you for personal information. Please provide answers only about women and men at the university in general. This discussion will be important for us to learn how to best address these issues. Are you ready to begin?

1. Let's start by talking about romantic relationships among university students. Tell me about relationships between male and female students.
2. Tell me about the sexual behaviors of students.
3. Do university students use alcohol or other substances?
4. Does using these substances affect their sexual behavior? How?
5. Now let's talk about how female students are treated on campus.
6. How do you define gender-based violence (GBV)?
7. How do you define intimate partner violence (IPV)?
8. Do these types of violence happen to female students at this university? Why?
9. Do male students ever do anything to discourage violence against women? What do they do?
10. What resources are available to female students on campus if they experience violence?
11. Does violence or the threat of violence affect female students' sexual health? How?
12. What happens to men at this university if they commit violence against a female student?
13. If incoming freshmen students were to receive training on sex and HIV, what would it look like?
14. What if incoming freshmen students were to receive training on gender-based violence, what would it look like?
15. Is there anything you wish the university would do differently for students regarding STIs/HIV? For male students in particular?
16. Is there anything you wish the university would do differently for students regarding GBV?
17. Is there anything else you would like to tell me about what we discussed today, or anything else you think I should know?
18. Do you have any questions for me?

Thank the respondents for their time, provide them with the resource guide, and deliver payment.
