# Supplementary material for: “We are responsible for the violence, and prevention is up to us”: a qualitative study of perceived risk factors for gender-based violence among Ethiopian university students
Source: BMC Womens Health. 2019 Nov 6;19:131. doi: 10.1186/s12905-019-0824-0 (PMC6836646; doi:10.1186/s12905-019-0824-0)
Supplement: Supplementary file 5 — Additional file 5. focus group guide for faculty/staff. [file 12905_2019_824_MOESM5_ESM.pdf]

Focus Group Discussion with FACULTY and STAFF

Thank you for agreeing to participate in the discussion. As I said during the consent process, I am going to ask you several questions about sex and violence on campus, including resources available to students. This discussion will be important for us to learn how to best address these issues. Are you ready to begin?

1. Let's start by talking about romantic relationships among university students. Tell me about relationships between men and women.
2. Tell me about the sexual behaviors of students.
3. Do university students use alcohol or other substances?
4. Are there any campus resources for students regarding sexual and reproductive health? Please describe them.
5. Now let's talk about how female students are treated on campus.
6. How do you define gender-based violence (GBV)?
7. How do you define intimate partner violence (IPV)?
8. Do these types of violence happen to female students at this university? Why?
9. Does violence or the threat of violence affect female students' sexual health? How?
10. Who do female students tell if they have experienced violence?
11. What resources are available to female students on campus if they experience violence?
12. What happens to men at this university if they commit violence against female students?
13. What is the response of the police or other legal bodies on campus regarding violence against female students?
14. Do female students ever come to you with gender-specific issues?
15. If incoming freshmen students were to receive training on sex and HIV, what
16. What if incoming freshmen students were to receive training on gender-based violence, what would it look like?
17. Is there anything you wish the university would do differently for students regarding STIs/HIV?
18. Is there anything you wish the university would do differently for students regarding GBV?
19. Is there anything else you would like to tell me about what we discussed today, or anything else you think I should know?
20. Do you have any questions for me?

Thank the respondents for their time and give them a copy of the resource guide.
